# Endovascular treatment of blunt injury of the extracranial internal carotid artery: the prospect and dilemma

**DOI:** 10.7150/ijms.50275

**Published:** 2021-01-01

**Authors:** Guangming Wang, Chao Li, Jianmin Piao, Baofeng Xu, Jinlu Yu

**Affiliations:** 1 Department of Neurosurgery, The First Hospital of Jilin University, Changchun, 130021, China; 2 Department of Neurology, The First Hospital of Jilin University, Changchun, 130021, China

**Keywords:** endovascular treatment, blunt injury, extracranial internal carotid artery

## Abstract

The extracranial internal carotid artery (ICA) refers to the anatomic location that reaches from the common carotid artery proximally to the skull base distally. The extracranial ICA belongs to the C1 segment of the Bouthillier classification and is at considerable risk for injury. Currently, the understanding of endovascular treatment (EVT) for blunt injury of the extracranial ICA is limited, and a comprehensive review is therefore important. In this review, we found that extracranial ICA blunt injury should be identified in patients presenting after blunt trauma, including classical dissection, pseudoaneurysm, and stenosis/occlusion. Computed tomography angiography (CTA) is the first-line method for screening for extracranial ICA blunt injury, although digital subtraction angiography (DSA) remains the “gold standard” in imaging. Antithrombotic treatment is effective for stroke prevention. However, routine EVT in the form of stenting should be reserved for patients with prolonged neurological symptoms from arterial stenosis or considerably enlarged pseudoaneurysm. Endovascular repair is now emerging as a favored therapeutic option given its demonstrated safety and positive clinical and radiographic outcomes.

## Introduction

**Figure 1:**
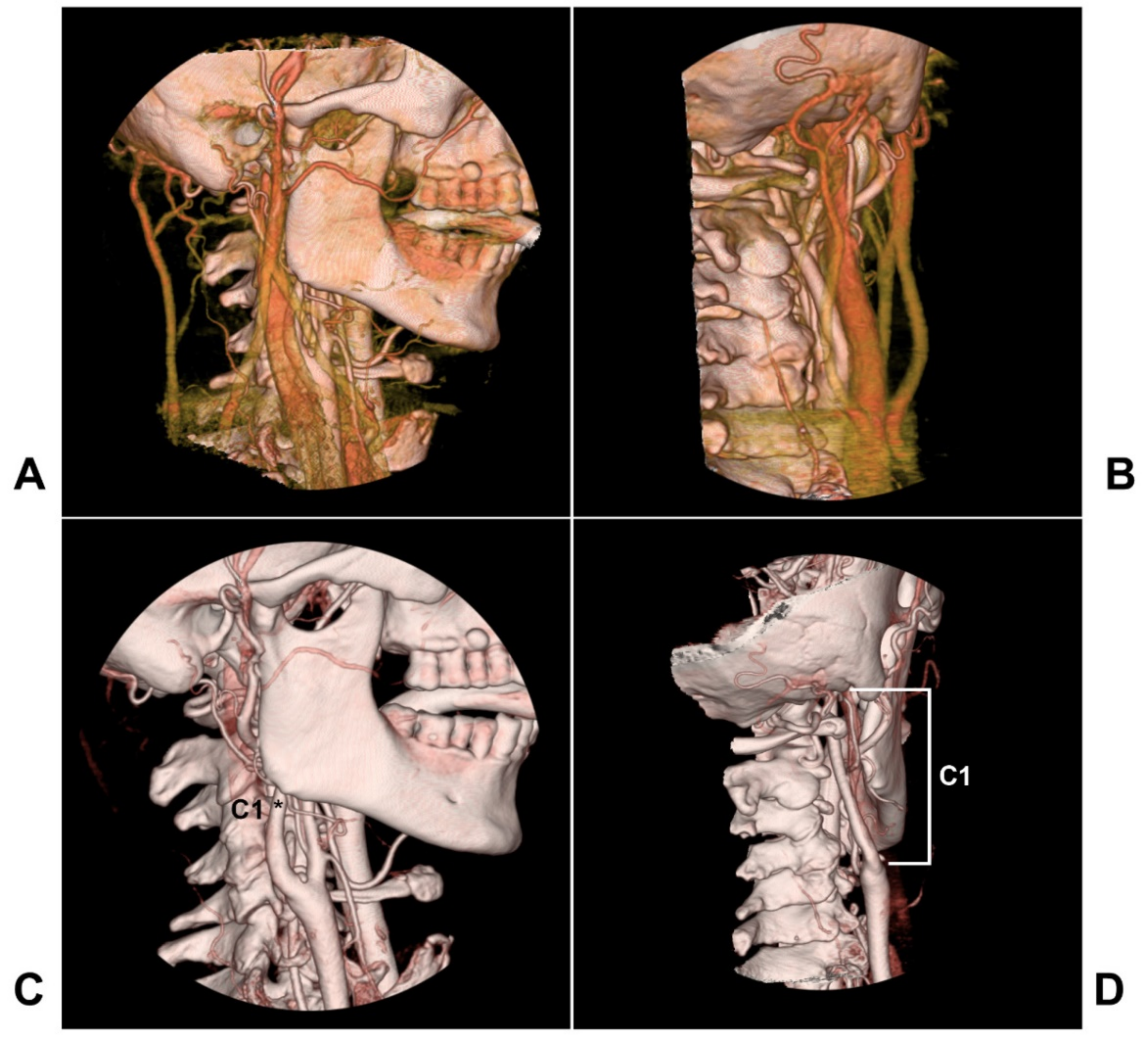
** The anatomy of the extracranial ICA region.** A-B: Lateral view (A) and posterior anterior view (B) of CTA showing carotid veins around the extracranial ICA. C-D: Lateral view (C) and posterior anterior view (D) of CTA showing that the extracranial ICA extends from the bifurcation to the skull base, and the extracranial ICA belongs to the C1 segment of the Bouthiller classification (asterisk). **Abbreviations**: CTA: computed tomography angiography; ICA: internal carotid artery

The extracranial internal carotid artery (ICA) refers to the anatomic location from the common carotid artery proximally to the skull base distally, and the segment ICA of the skull base is not included (Figure 1). In 1996, Bouthiller classified the ICA into seven segments: C1, cervical; C2, petrous; C3, lacerum; C4 cavernous; C5, clinoid; C6, ophthalmic; and C7, communicating 1. This classification is practical and clarifies all segments of the ICA, and the extracranial ICA therefore refers to the C1 segment.

Extracranial ICA is at high risk for injury because it is more mobile and vulnerable to stretching 2. The most common cause is blunt trauma from motor vehicle crashes, which results in a disruption in one or more layers of the extracranial ICA 3-5. Extracranial traumatic vascular injury is present in approximately 1% to 2% of patients after blunt trauma, and the incidence of extracranial ICA injury ranges from 0.08 to 0.33%, among which 52-79% of these patients are asymptomatic. For most of these patients, conservative medical treatment is sufficient 2, 6-10. However, for a small number of patients with disastrous implications, surgical intervention is needed 11.

Currently, endovascular treatment (EVT) has been used with encouraging outcomes 12, 13. However, the understanding of EVT for blunt injury of the extracranial ICA is limited, and a comprehensive review is therefore important. In this paper, a literature search was performed using the PubMed database and relevant search terms. After a review of the obtained literature, the current status of EVT for blunt injury of extracranial ICA was summarized in terms of pathogenesis, classification and grading, imaging examination, EVT prospects and controversies, EVT options, prognosis and complications.

## Pathogenesis

The blunt injury of the extracranial ICA begins with intimal damage resulting in dissection, which is the final common pathway for vascular injury. The dissection can be progressive and extend to the distal site, resulting in ICA stenosis/occlusion 14, 15.

When dissection disrupted almost all layers of the ICA wall with preservation of only the adventitia, pseudoaneurysm potentially occurred in 10% of cases with extracranial ICA blunt injury 6, 16. Pseudoaneurysms were defined as more than 50% focal dilatation of the ICA diameter and tended to occur in the upper cervical segment; moreover, they could grow 16, 17.

These injuries could be solitary or multiple, with one or more patterns of injury, and bilateral extracranial ICA could even be involved 9, 18-20.

Any kind of blunt injury of the extracranial ICA could result in platelet activation and thrombosis to cause distal embolization 21. In addition, when stenosis/occlusion occurs, the subsequent hemodynamics are compromised if collateral circulation is not satisfactory 6, 22. The majority of ischemic strokes occur relatively early; however, 17-36% of cases develop symptoms >24 h post injury, and 8% develop symptoms after a week 2, 9, 23.

## Classification and grading

For extracranial ICA blunt injury, there are several classifications, such as the Biffl scale, Borgess classification, and Seth supplemental classification to Biffl 24-27.

The Borgess classification includes four types. Type I dissections have an intact intima, with type IA displaying persistent antegrade flow and type IB demonstrating complete occlusion. Type II dissections have an intimal tear, with a small disruption of the intima and a small sidewall aneurysm in type IIA and a clear intimal flap and pseudoaneurysm in type IIB 24. However, for blunt carotid arterial injuries, the Borgess classification is limited.

Currently, the Biffl et al. scale is commonly utilized to assign vascular injuries and has become the standard for extracranial ICA blunt injury, from grade I to V as follows: grade I, irregularity or dissection with <25% stenosis; grade II, dissection with >25% luminal narrowing or a raised intimal flap; grade III, pseudoaneurysm; grade IV, complete occlusion; and grade V, ICA transection, active contrast extravasation 25, 26.

In this classification, grade V is very rare in extracranial ICA blunt injury because it is often caused by skull base fracture, which is not attributed to the extracranial ICA. Therefore, in our review, traumatic extracranial ICA transection was excluded, and only classical dissection, pseudoaneurysm, and traumatic stenosis/occlusion are discussed.

Furthermore, Seth et al. (2013) modified the Biffl et al. scale, further subdividing grade 2 and 3 injuries were further subdivided into 2a or 3a (non-flow-limiting when<70% luminal narrowing was present) and 2b or 3b (flow-limiting when >70% luminal narrowing was present) 27.

High grades had a greater risk of stroke; for instance, in the Biffl et al. (1999) study, the stroke rates with type I, type II, type III, and type IV ICA injuries were 3, 11, 33, and 44%, respectively 26. In addition, it must be emphasized here that follow-up imaging in extracranial ICA blunt injury is very important, as these injuries are variable; for instance, approximately 5% of low-grade injuries (88% pseudoaneurysms) can change to a higher grade 3, 28.

## Imaging examination

### Available imaging

Extracranial ICA blunt injury may be diagnosed using digital subtraction angiography (DSA), computed tomography angiography (CTA), magnetic resonance imaging/angiography (MRI/MRA), and ultrasonography; of these, ultrasonography alone is not an adequate test and is not recommended for screening, and DSA remains the “gold standard” 3, 29-33.

DSA can detect extracranial ICA blunt injury accurately with a sensitivity greater than 99% and a specificity of 100%; it also affords the opportunity to administer EVT 6, 33. However, this does not mean that DSA is preferred as the initial screening test 34.

Currently, the Eastern Association for the Surgery of Trauma's Blunt Cerebrovascular Injury Practice Management Guidelines recommend that CTA is the first-line method for screening for extracranial ICA blunt injury due to its increasing use and relative reliability 35.

MRI/MRA is most useful for evaluating patients with clinical evidence of stroke rather than as a screening tool 36. In addition, they are useful in the detection of dissections and intramural hematomas 37. Sometimes, routine computed tomography (CT) is also useful, as only CT can obtain secondary images of brain infarction and find high-density middle cerebral artery signs 38.

In addition, intraarterial optical coherence tomography is a new technique that provides in vivo evidence of arterial injury and thrombi not visible on CTA or DSA 39.

### Imaging characteristics

The imaging of extracranial ICA blunt injury varies as a result of various pathogeneses 40.

#### Classical dissection

Classical dissections include intramural hematomas, intimal tears and raised intimal flaps 8. Intramural hematomas present with eccentric/circumferential mural thickening extending along the long artery 18. Intimal tears present with subtle mural thickening and contour irregularity, and raised intimal flaps present with a linear pattern of filling defect/false lumen opacification known as the ''double lumen'' sign and even pseudofenestration 41.

#### Pseudoaneurysm

Pseudoaneurysms present with eccentric luminal dilatation and adjacent outpouchings 42, 43, and they are further classified into saccular and fusiform types 44. Saccular laminae have a distinct neck and a roundish dome and arise from a disruption of the elastic laminae 45. Fusiform laminae have a smooth, tapering shape and arise from a less extensive disruption of the elastic laminae 33. Saccular aneurysms have a greater tendency to enlarge than fusiform aneurysms 44.

#### Stenosis/occlusion

**Figure 2:**
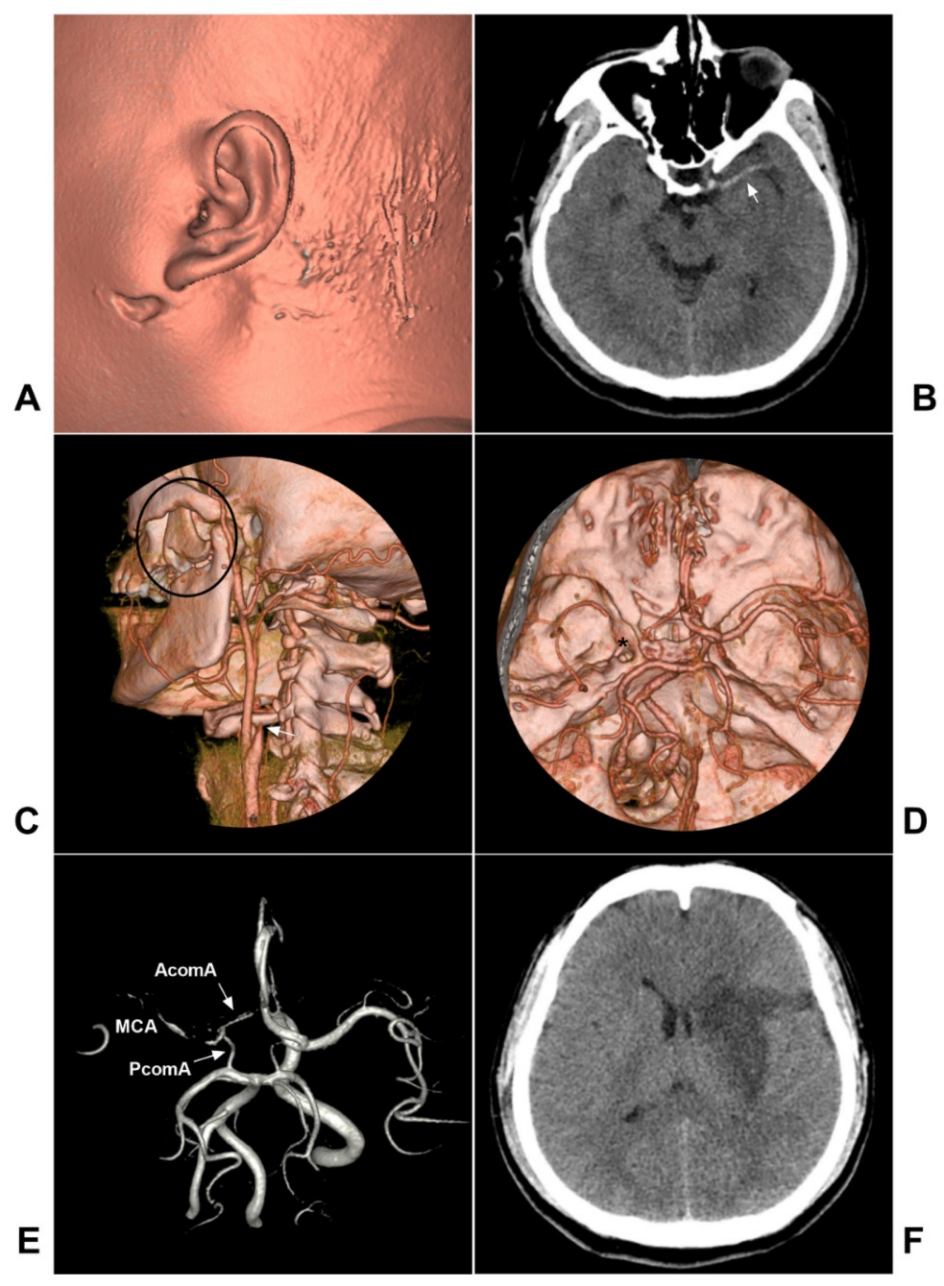
** Traumatic extracranial ICA occlusion.** A: CTA soft tissue reconstruction shows the left face injury in front of the ear. B: CT showed the left MCA high-density sign (arrow). C: CTA reconstruction showed maxillary bone fracture (black ellipse) and extracranial ICA occlusion (arrow). D: CTA reconstruction showed a thin intracranial MCA (asterisk). E: MRA showed the connections from AcomA and PcomA to MCA. F: Follow-up CT showed infarction of the left basal ganglia region. **Abbreviations**: AcomA: anterior communicating artery; CT: computed tomography; CTA: CT angiography; ICA: internal carotid artery; MCA: middle cerebral artery; MRA: magnetic resonance angiography; PcomA: posterior communicating artery

Dissection-related stenosis usually appears as a smooth and tapered narrowing that varies in severity and length depending on the extent of the dissection, and the occlusion demonstrates a tapered, flame-like appearance 15. On DSA, antegrade and retrograde collateral obstruction channels, including the anterior and posterior communicating arteries and ophthalmic artery, can be seen in the acute phase of arterial occlusion, and rapid assessment of collateral circulation on DSA is thus necessary 46, 47. A typical case is shown in Figure 2.

## EVT prospects and controversy

### EVT prospect

Low-grade extracranial ICA blunt injury should be treated with early systemic anticoagulation or antiplatelet therapy 6, 48-51. The Eastern Association for the Surgery of Trauma recommends against the routine use of endovascular stents as an adjunct to antithrombotic therapy in patients with asymptomatic grade II or stable III extracranial ICA blunt injuries 52, 53.

For flow-limiting dissections of grade II, balloon angioplasty or stenting is used 54. For expanding or symptomatic pseudoaneurysms (grade III) (depth>15 mm), stenting with/without coiling is necessary 55, 56. For vessel occlusion (grade IV) with acute flow-related infarcts, mechanical thrombectomy or recanalization EVT may be considered to restore flow 57.

### EVT dilemma

For severe and complex extracranial ICA blunt injuries, EVT reconstruction is sometimes difficult and fails, thus causing several pitfalls, including microcatheterization of the true carotid lumen 27. In addition, microcatheterization may worsen the dissection or release thrombi, or the microcatheter may accidentally remain in the false lumen 58, 59.

For some extracranial ICA blunt injuries, arterial occlusion is sometimes the last resort 11, 60. However, when performing arterial occlusion, the balloon occlusion test (BOT) is not absolutely safe, as it is not reliable for semicomatose patients 61, 62. In addition, patients who passed the BOT can exhibit ischemic events 63-65. Moreover, arterial occlusion carries a risk of migration of the intraluminal thrombus into the intracranial circulation 66.

In addition, for extracranial ICA blunt injury, EVT using stents requires antiplatelet therapy, which can be problematic in trauma patients. If the antiplatelet agents are not used routinely, acute thrombosis occurs in 45% of patients; thus, it seems prudent to limit EVT 11.

## Endovascular option

### Outline

EVT for extracranial ICA blunt injury can be divided into reconstruction and deconstruction techniques 2.

### Reconstruction

Reconstructive EVT includes mechanical thrombectomy, balloon angioplasty, traditional carotid artery stenting (CAS) and flow-diverting stent (FDS) deployment, and intracranial stent deployment 67, 68.

#### Mechanical thrombectomy

If thrombosis forms in the ICA true lumen after blunt injury, endovascular clot removal is an attractive option for the treatment of acute stroke in the early stage; however, the clot is often dissected, and it is difficult or dangerous to remove the intramural hematoma 2, 69. In addition, mechanical thrombectomy should be used in the stenting combination 70.

#### Carotid artery stent deployment

For symptomatic dissection, CAS allows better containment of the mural thrombus between the stent and vessel wall, with a >99% technique success rate 2, 71. During CAS, the stent should cross the entire length of the dissection; when using overlapping stents, the initial stent should be placed at the proximal margin of the dissection 27, 72. Currently, CAS is the mainstay for most proximal and middle extracranial ICA dissections 55.

**Figure 3:**
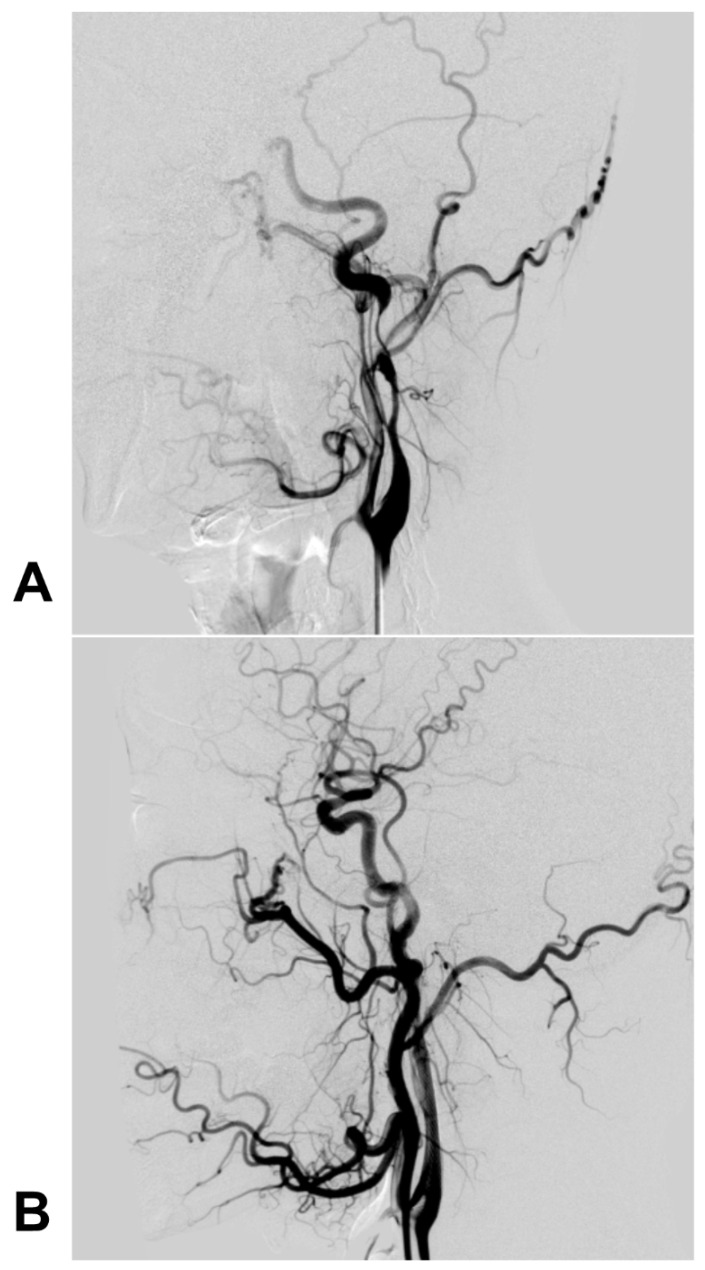
** Carotid stent deployment for traumatic extracranial ICA dissection with severe stenosis.** A: The preoperative DSA revealed a long dissection with severe stenosis of the extracranial ICA, and the intracranial blood supply was poorly visible. B: DSA after carotid stenting showed that the extracranial ICA recovered to a normal diameter, and the intracranial blood supply was normal. **Abbreviations**: DSA: digital subtraction angiography; ICA: internal carotid artery; MRA: magnetic resonance angiography; MCA: middle cerebral artery; PcomA: posterior communicating artery

For pseudoaneurysms, CAS with/without coil embolization of the aneurysm cavity is a feasible technique 73. For instance, Kadkhodayan et al. (2005) reported that 9 traumatic carotid dissections with/without associated pseudoaneurysms were effectively treated with CAS; in this report, some pseudoaneurysms were given coiling simultaneously 71. Currently, there is no consensus on whether coiling is needed during CAS for pseudoaneurysms, but it is reasonable that large and wide-necked coiling should be recommended 74-76. A typical case is shown in Figure 3.

#### Covered stent deployment

By setting up a direct physical barrier, the covered stent allows the immediate closure of the leakage while maintaining the patency of the parent artery; for extracranial ICA pseudoaneurysm, the covered stent graft has appeal 43, 67, 77. In Wang et al. (2020), 5 patients with traumatic carotid pseudoaneurysms underwent covered stenting, and complete exclusion of the pseudoaneurysm and patency of the parent artery were maintained during follow-up 78.

**Figure 4:**
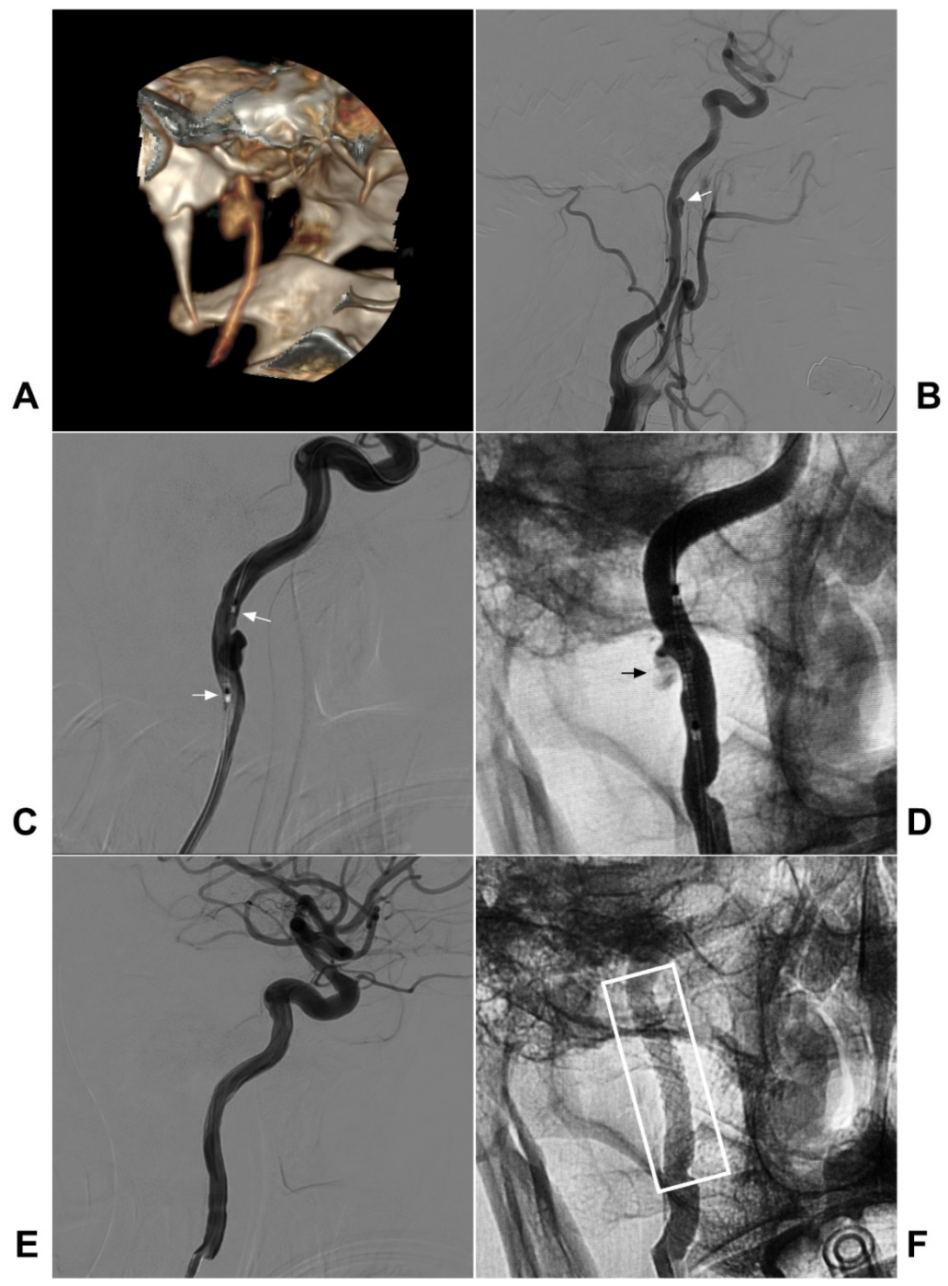
** Covered stent deployment for the traumatic extracranial ICA pseudoaneurysm.** A-B: CTA (A) and DSA (B) showed a pseudoaneurysm in the high segment extracranial CIA (arrow in B). C: A covered stent (MicroPort Medical Co., Shanghai, China) crossed the pseudoaneurysm (arrows show the distal and proximal markers). D: After covered stent deployment, endoleak was observable (arrow). E: After the balloon dilation was repeated, the endoleak disappeared. F: X-ray showing the covered stent (white frame). **Abbreviations**: CTA: CT angiography; DSA: digital subtraction angiography; ICA: internal carotid artery

During covered stent deployment, to ensure good adherence and avoid endoleak, the stent diameter is selected mainly according to the larger diameter of the proximal and distal ICA, and the length of the stent must be long enough 42, 79. A typical case is shown in Figure 4.

#### Flow-diverting stent deployment

The FDS is a self-expanding tubular mesh made of cobalt-chromium with more than 30% metal coverage and 3 times the vessel wall coverage compared with traditional intracranial stents 80. The woven tubes can provide a significantly increased potential for thrombosis by diverting blood flow away from the aneurysm and reconstructing the diseased parent artery as well as providing a scaffold for endothelial healing 81.

It is difficult to access extracranial ICA blunt injuries with the inflexible carotid stent delivery system when they are located in the high extracranial ICA near the skull base because of ICA tortuosity 82. In contrast, the FDS is flexible, enabling the device to more efficiently conform to vessel curves 83.

Therefore, the FDS should be suitable for treating distal extracranial ICA dissections and pseudoaneurysms 55. For instance, Brzezicki et al. (2016) reported 13 extracranial ICA dissections, of which 9 were caused by trauma. After FDS deployment, complete revascularization was achieved in 91% of vessels, and 50% of pseudoaneurysms were completely or near completely obliterated immediately 84.

With FDS deployment, a higher risk of FDS migration may occur in the extracranial ICA than in intracranial vessels because of the high luminal pressure and frequent positional changes with neck movement; thus, proximal migration of the FDS is oversized relative to that of the extracranial ICA 81. For some extracranial ICA injuries, CAS can be performed after FDS deployment because the FDA can easily pass the complex and tortuous ICA segment 85.

In addition, multiple overlaying FDSs can be used when necessary 81.

#### Intracranial stent deployment

Although intracranial stents can be used for this indication, they may not possess enough radial force to exclude false lumen or provide enough flow diversion to promote thrombosis of the pseudoaneurysm; because of their large cell design, they can rarely be used alone 74. Therefore, the intracranial stent cannot be used routinely 86.

However, if coiling is combined, it may be a good choice 87. For instance, Joo et al. (2016) successfully treated an extracranial ICA pseudoaneurysm with stenting and coiling, and the EVT was satisfactory after a one-year follow-up 88.

### Deconstruction

For extracranial ICA blunt injuries that cannot be treated by stenting reconstruction, such as some severe pseudoaneurysms, therapeutic arterial occlusion must be performed 16. Certainly, the BOT must be performed before considering this option 89. For arterial occlusion, the embolic materials include detachable balloons, coils, and liquid embolic agents 90, 91.

## Prognosis and complication

For extracranial ICA blunt injury, the prognosis can be judged by the modified Rankin Scale (mRS), and poor outcome is defined as recurrent stroke or a mRS score of 2 or more 48.

In general, for extracranial ICA blunt injury, the survivors who accept EVT have a good prognosis due to the high success rate 92, 93. The systematic review of Pavlos et al. (2020) included twenty-four studies comprising 191 patients (204 lesions) and showed that EVT for extracranial ICA blunt injury was associated with excellent outcomes and efficiently prevented recurrent stroke and TIA and demonstrated excellent recanalization and pseudoaneurysm occlusion rates with very low retreatment rates 94.

Complications of EVT in extracranial ICA blunt injury are low 95. In a retrospective study, Kadkhodayan et al. (2005) demonstrated a complication rate of 6.9% 71. The complications of EVT in extracranial ICA blunt injury were associated with injury grading or the EVT procedure. For instance, for extracranial ICA blowouts, Choi et al. (2018) reported a procedure-related complication rate of 37.5% and a cerebral infarction rate of 25% 96.

## Conclusion

Extracranial ICA blunt injury should be identified in patients presenting after blunt trauma, including classical dissection, pseudoaneurysm, and stenosis/occlusion. CTA is the first-line method for screening for extracranial ICA blunt injury. Antithrombotic treatment is effective for stroke prevention. However, routine EVT in the form of stenting should be reserved for patients with prolonged neurological symptoms from arterial stenosis or considerably enlarging pseudoaneurysm. Endovascular repair is now emerging as a favored therapeutic option given its demonstrated safety and positive clinical and radiographic outcomes.
